# Lysimachiae Herba Modulates FXR to Alleviate Cholestatic Liver Injury: Insights from Serum Pharmacochemistry and Experimental Validation

**DOI:** 10.3390/cimb48070682

**Published:** 2026-07-02

**Authors:** Wei Zhao, Bao Yu, Chengli Li, Jingjing Li, Haijun Huang, Weiguo Cao

**Affiliations:** 1 School of Chinese Materia Medica, Chongqing University of Chinese Medicine, Chongqing 402760, China; zw010814@163.com (W.Z.); yubao@cqctcm.edu.cn (B.Y.); lijingjing@cqctcm.edu.cn (J.L.); 2 College of Traditional Chinese Medicine, Chongqing Medical University, Chongqing 400016, China; niubei879@163.com

**Keywords:** Lysimachiae Herba, cholestatic liver injury, RNA sequencing, bile acid homeostasis, traditional Chinese medicine

## Abstract

Cholestatic liver injury (CLI) is a complex condition for which current treatment options remain limited. Lysimachiae Herba (LH), a traditional Chinese medicine with hepatoprotective properties, has not yet been fully characterized in terms of its active constituents and underlying mechanisms in CLI. This study was designed to systematically determine the chemical composition of LH, characterize its absorbed constituents in vivo, and elucidate its therapeutic mechanisms against CLI. UPLC-Q-TOF-MS/MS was employed to analyze the chemical composition of LH and its absorbed components in rat serum. Key targets and signaling pathways were predicted using network pharmacology and molecular docking, followed by experimental validation in an ANIT-induced CLI mouse model and LCA-treated HepG_2_ cells through biochemical assays, histological examination, transcriptomic analysis, qRT-PCR, Western blotting, and immunofluorescence analysis. A total of 129 compounds were tentatively identified in LH, among which 26 were detected in the bloodstream. Network analysis and molecular docking suggested that LH regulates bile acid homeostasis predominantly by the FXR signaling pathway. Both in vivo and in vitro experiments provided convergent evidence that LH modulates the FXR-related bile acid regulatory network, enhances bile acid efflux transporter expression, and alleviates CLI. In conclusion, this study systematically elucidates the chemical composition, absorbed constituents, and pharmacological mechanisms of LH in CLI, highlighting the involvement of FXR-related bile acid regulation as an important mechanism and providing a scientific basis for the potential development of LH for cholestatic liver injury.

## 1. Introduction

Cholestatic liver injury is typified by impairment of bile formation, secretion, or flow, leading to bile accumulation within hepatocytes, intrahepatic ductal damage, and portal inflammation [1]. CLI occurs in a variety of cholestatic liver diseases, including primary biliary cholangitis (PBC), primary sclerosing cholangitis (PSC), drug-induced cholestasis, intrahepatic cholestasis of pregnancy, biliary atresia, and obstructive cholestasis [2]. PBC is mainly marked by granulomatous injury targeting small intrahepatic bile ducts, whereas PSC is characterized by chronic inflammation and fibrosis of the intrahepatic and extrahepatic bile ducts, leading to multifocal bile duct strictures [3]. Although ursodeoxycholic acid (UDCA) remains the first-line therapy for PBC, a considerable proportion of patients show an inadequate biochemical response. Moreover, effective pharmacological treatment options for PSC remain limited [4,5]. Therefore, it remains necessary to explore new therapeutic agents and mechanisms for the prevention and treatment of CLI.

Bile acid (BA) accumulation is not only a biochemical feature of cholestasis but also an important driver of liver injury. Excessive intrahepatic BAs can induce hepatocyte toxicity, oxidative stress, mitochondrial dysfunction, and inflammatory responses, thereby aggravating cholestatic liver damage [6]. Farnesoid X receptor (FXR), a nuclear BA receptor, plays a central role in maintaining BA homeostasis by regulating BA synthesis, uptake, and efflux through downstream targets such as SHP, CYP7A1, CYP27A1, NTCP, and BSEP [7]. Accordingly, FXR-mediated BA regulation has been considered an important therapeutic strategy for cholestatic liver diseases.

Lysimachiae Herba (LH), the dried whole herb of *Lysimachia christinae* Hance, is a traditional Chinese medicine commonly employed for hepatobiliary conditions such as jaundice, gallstones, and cholestasis [8,9]. Previous pharmacological studies have demonstrated that LH exhibits hepatoprotective, anti-inflammatory, and bile-regulating activities [10]. Phytochemical investigations have shown that LH is rich in various bioactive compounds, including phenolic acids, flavonoids, and terpenoids, which are considered to contribute to its therapeutic effects [11,12]. Nevertheless, owing to the intrinsic complexity of traditional Chinese medicine, the bioactive constituents and molecular mechanisms responsible for the anti-cholestatic effects of LH remain poorly understood.

In recent years, integrated analytical strategies combining advanced chemical profiling and systems biology approaches have provided powerful tools for deciphering the pharmacological mechanisms of complex herbal medicines. High-resolution mass spectrometry combined with ultra-performance liquid chromatography allows for sensitive and accurate profiling of chemical components [13]. Serum pharmacochemistry further focuses on identifying the bioavailable components that enter systemic circulation, thereby providing a more realistic representation of the bioavailable constituents that may contribute to in vivo efficacy [14]. In addition, network pharmacology provides a holistic approach to understanding the multi-component, multi-target, and multi-pathway actions of herbal medicines, while molecular docking allows for the prediction of binding strengths between bioactive compounds and target proteins [15,16,17].

In this study, we integrated UPLC-Q-TOF-MS/MS-based chemical profiling, serum pharmacochemistry, network pharmacology, molecular docking, transcriptomic analysis, and experimental validation to systematically investigate the chemical constituents, blood-absorbed components, and potential mechanisms of LH against CLI. The research design followed a stepwise strategy from chemical characterization and blood-absorbed component identification to target prediction, pathway screening, and biological validation. This study aimed to investigate whether FXR-related BA homeostatic regulation is involved in the protective effects of LH against CLI and to provide a scientific basis for its further development in cholestatic liver diseases.

## 2. Materials and Methods

### 2.1. Chemicals and Reagents

1-naphthyl isothiocyanate (ANIT, 551-07-5) was supplied by Adamas (Shanghai, China). Lithocholic acid (LCA; purity: HPLC ≥ 97%, L106880-1g) was obtained from Aladdin Biochemical Technology Co. Ltd. (Shanghai, China). Ursodeoxycholic acid capsules (UDCA) were obtained from Losan Pharma GmbH (Neuenburg am Rhein, Germany). Olive oil (8001-25-0) was purchased from Greagent (Shanghai, China). Alkaline phosphatase (ALP, A059-2), alanine aminotransferase (ALT, C009-2-3), total bile acid (TBA, E003-1-3) and aspartate aminotransferase (AST, C010-3-2) were purchased from Nanjing Jiancheng Bioengineering Institute (Nanjing, China). Total bilirubin (TBIL, JL-T1397) was supplied by JONLNBIO (Shanghai, China). SYBR Green Premix Pro Taq HS qPCR Kit and Evo M-MLV RT Mix Kit with gDNA Clean for qPCR Ver.2 were supplied by Accurate Biology (Changsha, China). RNA isolater Total RNA Extraction Reagent (Trizol) was provided by Vazyme (Nanjing, China). HRP-conjugated Goat Anti-Rabbit IgG was provided by BIONIKY (Shanghai, China). The Anti-SHP Antibody (SHP, SN72-02), Anti-CYP27A1 Antibody (CYP27A1, JG40-36), Anti-SLC10A1 Antibody (NTCP, ER1916-66) and Anti-FXR Antibody (FXR, ER1909-14) were procured from HUABIO (Hangzhou, China); CYP7A1 Polyclonal antibody (CYP7A1, 18054-1-AP), BSEP Monoclonal antibody (BSEP, 67512-1-Ig), GAPDH Monoclonal antibody (GAPDH, 60007-2-Ig) and HRP-conjugated Goat Anti-Mouse IgG (SA00002-2) were supplied by Proteintech (Wuhan, China).

### 2.2. Plant Material, Authentication, and Extract Preparation

The whole plant material of *Lysimachia christinae* Hance, harvested from Nanchuan, Chongqing on 3 December 2024 was authenticated by Professor Kaizhi Hu. The voucher specimen (No. 202412) was archived in the Chongqing Key Laboratory of Basic and Applied Research in Bayu Formulas and Medicinals, Chongqing. The dried LH herb was mixed with ultrapure water at a ratio of 1:12 (*w*/*v*) and soaked overnight. The mixture was then heated for 2 h and filtered. The residue was re-extracted with ultrapure water (1:8, *w*/*v*) for 1 h. The combined filtrates were concentrated, evaporated, and finally freeze-dried to obtain a powdered extract for subsequent compound identification and animal experiments.

### 2.3. Animal Experiments

Forty-eight male SPF-grade C57BL/6J mice (18–22 g) were acclimatized for 7 days and subsequently randomized into six groups with eight mice in each group. The Institutional Animal Care and Use Committee of Chongqing Medical University (IACUC-CQMU) approved all animal experiments described in this study. The mice were randomly divided into six groups: Control group, ANIT (40 mg/kg) group, ANIT + UDCA (90 mg/kg) group, ANIT + LH with low dose (LH-L, 2 g/kg) group, ANIT + LH with medium dose (LH-M, 4 g/kg) group and ANIT + LH with high dose (LH-H, 8 g/kg) group. The control and ANIT model groups received purified water by daily gavage for 12 days. All other groups were administered their respective drugs at an administration volume of 0.1 mL/10 g body weight daily for the same period. To induce CLI, mice in all groups except the control were additionally gavaged with ANIT (40 mg/kg) on days 1, 4, 7, and 10, after which the designated drug or vehicle treatments resumed. Following the final administration, mice underwent a 12 h fasting period before being euthanized. Blood and liver specimens were obtained for later analysis.

Six male SPF-grade Sprague–Dawley rats (200–220 g) were divided into two groups: a vehicle control group and an LH group (10 g/kg). All rats received daily gavage for 7 days, with the control group administered distilled water and the LH group administered the LH solution. Following the final administration, orbital sinus blood was obtained at six time points (0, 30, 60, 120, 240, and 480 min). Serum from each time point was pooled, centrifuged, and the resulting supernatant was used for subsequent analysis of serum-absorbed constituents.

### 2.4. Serum Biochemical Analysis

Mouse serum samples were obtained by centrifugation at 12,000 rpm for 15 min at 4 °C, and the concentrations of TBIL, ALT, TBA, ALP and AST were measured using commercial assay kits following the procedures provided by the manufacturers.

A mixture of 200 μL rat serum and 1 mL methanol was vortexed. Following centrifugation at 12,000 rpm for 15 min, the supernatant was carefully transferred to a fresh Eppendorf tube and evaporated to dryness under nitrogen gas at room temperature. The residue was reconstituted in 100 μL of methanol, recentrifuged, and the resulting supernatant was collected for subsequent instrumental analysis.

### 2.5. Histological Examination of Liver Tissue

Following fixation in 4% paraformaldehyde, liver tissues were processed through dehydration steps and embedded in paraffin. Liver tissue sections (4 μm thick) were prepared, stained with hematoxylin-eosin (H&E), and then examined histologically using a Panoramic SCAN digital slide scanner (3DHISTECH, Budapest, Hungary). Slide Viewer 2.5.0 software was used to capture images for subsequent histological documentation and analysis.

### 2.6. UPLC-Q-TOF-MS/MS Analysis of LH Extract and Serum Samples

UPLC-Q-TOF-MS/MS was used to characterize both the chemical constituents of the LH extract and the blood-absorbed components in rat serum after intragastric administration of LH. The analysis of the LH extract was performed to establish the overall chemical profile of the herbal preparation, whereas serum pharmacochemistry was conducted to identify the constituents that entered the systemic circulation and therefore were more likely represented the bioavailable material basis of LH. The identified constituents in the LH extract are listed in Supplementary Table S1.

### 2.7. UPLC and Mass Spectrometry Conditions

UPLC-Q-TOF-MS/MS analysis was performed using an Agilent 6546 LC/Q-TOF mass spectrometer. Chromatographic separation was performed using an InfinityLab Poroshell 120 SB-C18 column (2.1 × 100 mm, 2.7 μm; Agilent). Gradient elution was carried out with solvent A (0.1% formic acid in water) and solvent B (0.1% formic acid in acetonitrile), following the program described below: 0–0.5 min, 0–5% B; 0.5–13 min, 5–30% B; 13–20 min, 30–60% B; 20–25 min, 60–75% B; 25–29 min, 75–95% B; 29–31 min, 95–5% B. The flow rate was set at 0.3 mL/min, and the injection volume was 5 μL, with the column temperature held constant at 30 °C. Mass spectrometry data were acquired in both positive and negative ESI modes using data-dependent acquisition (DDA) across an *m*/*z* range of 100–1500. The instrument settings were as follows: drying gas temperature 320 °C, nitrogen drying gas flow 8.0 L/min, nebulizer pressure 35 psi, OCT RF voltage 750 V, capillary voltage 3500 V, and fragmentor voltage 175 V. Data acquisition and analysis were carried out using Agilent MassHunter Workstation software. For serum pharmacochemistry, blank serum samples were analyzed in parallel to exclude endogenous components and background signals. Only peaks detected in LH-treated serum but absent or markedly lower in blank serum were considered potential absorbed constituents.

### 2.8. Cholestasis Target-Component Collection and Network Construction

The keyword “cholestasis” was used for retrieval, and the corresponding gene targets of cholestasis were gathered by searching three databases: Online Mendelian Inheritance in Man (OMIM, http://omim.org, accessed on 1 December 2025), Therapeutic Target Database (TTD, http://db.idrblab.net, accessed on 1 December 2025), and GeneCards (http://www.genecards.org, accessed on 1 December 2025). After removing duplicate and redundant entries, the targets integrated from these databases were acquired. The Swiss Target Prediction (https://swisstargetprediction.ch/, accessed on 1 December 2025) was used for predicting the targets of the serum-absorbed components in rats. The Drug–Disease–Ingredient–Target (DDIT) network was constructed using Cytoscape (v3.7.0) software. Network topology analysis was then performed with the built-in “Network Analyzer” tool to identify core candidate constituents.

### 2.9. Protein–Protein Interaction Network

The STRING database (https://string-db.org, accessed on 1 December 2025) was utilized to import the intersecting targets, thereby establishing a protein–protein interaction (PPI) network between LH compound-derived targets and cholestasis-associated targets. A high confidence score of 0.7 was applied to filter for high-confidence interactions. The targets derived from the STRING database were then uploaded to Cytoscape (v3.7.0), with the PPI network constructed according to the output of NetworkAnalyzer (version 3.8).

### 2.10. GO Enrichment Analysis and KEGG Pathway Analysis

GO (Gene Ontology) biological process annotation and KEGG (Kyoto Encyclopedia of Genes and Genomes) pathway analysis of the targets were carried out using the DAVID database (https://David.Ncifcrf.Gov/summary.JSP, accessed on 1 December 2025). Based on the fold enrichment value, the top 10 or 20 entries were selected to elucidate the signaling pathways and molecular mechanisms underlying the therapeutic effect of LH against cholestasis.

### 2.11. Molecular Docking Studies

To acquire structural information, the protein structures of the critical targets were retrieved from PDB (https://www.rcsb.org/, accessed on 1 December 2025), and the structural data of the core bioactive constituents were extracted from the TCMSP database (https://www.tcmsp-e.com/index.php, accessed on 1 December 2025). To identify active pockets suitable for docking, the target proteins and small-molecule compounds were first prepared. Proteins were processed with PyMOL (version 3.1.6.1), while small molecules were handled with Chem3D (version 22.2.0), reconfigured into standard formats, and analyzed to map their protein-binding sites. Following the import of preprocessed target proteins and formatted small-molecule compounds into AutoDockTools software (version 4.2.6), the coordinates of the docking sites were defined, and the docking process was validated. The binding affinity between the aforementioned small molecule compounds and target proteins was analyzed, and the docking conformation with the lowest binding energy was selected for further analysis and visualization using PyMOL.

### 2.12. RNA Sequencing Analysis

The RNA-sequencing (RNA-seq) assay was carried out by Majorbio (Shanghai, China). RNA-seq analysis was performed on liver tissues from the Control, ANIT, and LH-H groups, with three biological replicates per group. Mouse liver tissue total RNA was extracted, and the integrity of the extracted RNA was rigorously assessed. After completing library construction and passing quality control, the qualified total RNA was subjected to transcriptome analysis using the Illumina NovaSeq platform. The RNA-seq results were subjected to data quality control, reference genome mapping, and quantitative analysis. The DESeq2 (version 3.23) software package was utilized for the identification of genes with differential expression among three groups. GO enrichment analysis of differentially expressed genes (DEGs) was performed using the Goatools toolkit. For KEGG pathway enrichment, the Python 3.11.0 scipy package was applied. A corrected *p*-value below 0.05 was set as the threshold for statistical significance.

### 2.13. Cell Culture and Treatment

HepG_2_ cells were grown in Minimum Essential Medium (MEM) supplemented with non-essential amino acids (NEAA), 10% FBS, 100 U/mL penicillin G, and 100 μg/mL streptomycin sulfate, while maintaining a 37 °C, 5% CO_2_ humidified environment. HepG_2_ cells were divided into the control group, LCA (100 μM) group, LCA + LH with low dose (LH-L, 25 μg/mL) group, LCA + LH with medium dose (LH-M, 50 μg/mL) group and LCA + LH with high dose (LH-H, 100 μg/mL) group. Following a 24 h incubation period, the expression levels of target proteins were assessed by Western blot and immunofluorescence analysis.

### 2.14. qRT-PCR Analysis

RNA was extracted from liver samples using Trizol reagent. Subsequently, cDNA was synthesized with the Evo M-MLV RT Mix Kit in a BIO-RAD T100™ Thermal Cycler (Los Angeles, CA, USA). Quantitative PCR was then performed on a CFX Connect™ Real-Time System (BIO-RAD,Los Angeles,, CA, USA) using the SYBR Green Premix Pro Taq HS qPCR Kit under standard thermal cycling conditions. The resulting data were normalized to GAPDH as an internal control and quantified using the 2^−ΔΔCt^ method. The specific primers used for the target genes are listed in Table 1.

### 2.15. Western Blot Analysis

Liver tissues or HepG_2_ cells were lysed in RIPA buffer with 1% PMSF, and after centrifugation, the supernatants were collected for protein quantification using a BCA kit. Following mixing with loading buffer and heat denaturation, proteins were separated by SDS-PAGE, transferred to PVDF membranes, and then blocked and washed. The membranes were incubated overnight at 4 °C with primary antibodies (FXR, SHP, CYP7A1, NTCP, CYP27A1, BSEP, and GAPDH), followed by secondary antibody application, with band visualization performed on the ODYSSEY FC system (LI-COR, USA). ImageJ 1.54 was used for band intensity measurement, and all signals were normalized to GAPDH.

### 2.16. Immunofluorescence

Following a 24 h incubation period, cells were fixed with 4% paraformaldehyde for 20 min, followed by permeabilization with 0.5% Triton X-100 in PBS for 30 min at room temperature. Prior to overnight incubation with the primary antibody at 4 °C, the cells were blocked with 5% BSA in PBS for 1 h; thereafter, a goat anti-rabbit fluorescent secondary antibody was applied for 2 h at room temperature in the dark. DAPI was then used to stain the nuclei under light-protected conditions. Images were captured using a Leica fluorescence microscope (Wetzlar, Germany).

### 2.17. Statistical Analysis

All statistical analyses were performed using GraphPad Prism 10.1.2 (GraphPad Software Inc., San Diego, CA, USA). Data are presented as mean ± standard error of the mean (SEM) for each experimental group. For comparisons, one-way ANOVA was applied. Statistical significance against the control group is indicated as ^#^ *p* < 0.05, ^##^ *p* < 0.01, and ^###^ *p* < 0.001, while significance versus the model group is shown as * *p* < 0.05, ** *p* < 0.01, and *** *p* < 0.001.

## 3. Results

### 3.1. Component Identification of LH and Analysis of Serum-Absorbed Constituents

**Figure 1:**
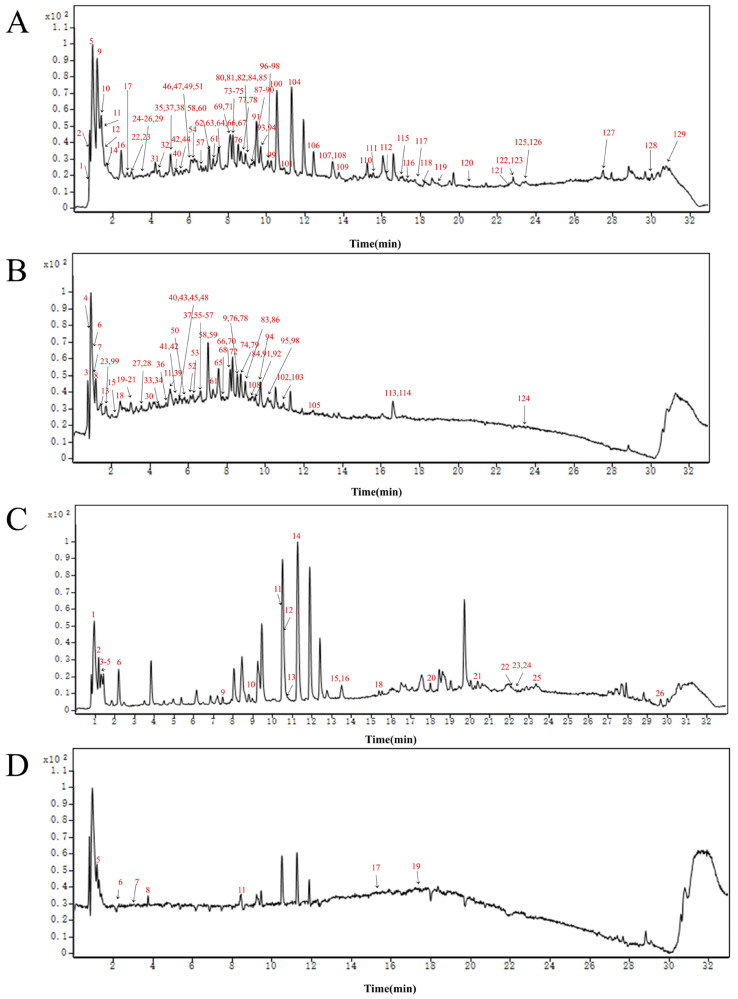
Total ion chromatograms of LH extract and serum-absorbed constituents after oral administration of LH. (**A**) Positive-ion mode TIC of LH extract. (**B**) Negative-ion mode TIC of LH extract. (**C**) Positive-ion mode TIC of rat serum after LH administration. (**D**) Negative-ion mode TIC of rat serum after LH administration.

To clarify the overall chemical composition of LH and its bioavailable constituents, UPLC-Q-TOF-MS/MS analysis was separately performed on the LH extract and rat serum samples collected after oral administration of LH. A total of 129 chemical constituents (Figure 1A,B) were tentatively identified in the LH extract by comparing accurate masses, retention times, MS/MS fragment ions, adduct ions, database information, and literature data [18,19,20]. The detailed information is provided in Supplementary Table S1. These constituents mainly included phenolic acids, flavonoids, terpenoids, phenylpropanoid-related compounds, amino acids, and other small molecules. Representative phenolic acids and phenylpropanoid-related constituents included p-coumaric acid, 2-hydroxycinnamic acid, ferulic acid, gallic acid, protocatechuic acid, syringic acid, and caffeic acid derivatives. Representative flavonoids and flavonoid glycosides included genistein, epicatechin, myricitrin, kaempferide, isoschaftoside, rutin, quercetin derivatives, and kaempferol derivatives. These findings are consistent with the phytochemical characteristics of LH, which is rich in phenolic acids and flavonoids.

After oral administration of LH, 26 serum-absorbed constituents (Figure 1C,D) were detected in rat serum and are summarized in Table 2. Most of these constituents could be matched with corresponding peaks in the LH extract based on molecular formula, accurate mass, retention behavior, MS/MS fragmentation, and adduct ions, indicating that they were mainly prototype constituents absorbed from LH [21,22]. These serum-absorbed constituents included phenolic acids and related compounds, such as p-coumaric acid, 2-hydroxycinnamic acid, gallic acid, protocatechuic acid, ferulic acid, and syringic acid; flavonoids, such as genistein, epicatechin, myricitrin, kaempferide, and isoschaftoside; and other constituents, such as 10-gingerol, L-menthol, nardosinone, undulatoside A, a homoisoflavanone derivative, dimethylophiopogonanone, and 2-methoxycinnamaldehyde. In addition, several small molecules and amino acids, including valine, isoleucine, phenylalanine, and tryptophan, were also detected in serum. Because these compounds may also be related to endogenous rat metabolism, they were interpreted cautiously and were not considered specific plant-derived markers alone. Overall, the comparison between the LH extract and serum samples narrowed the broad chemical profile of LH from 129 extract constituents to 26 blood-exposed constituents, providing a more bioavailability-oriented chemical basis for subsequent network pharmacology and mechanistic validation.

### 3.2. Serum-Absorbed Constituents and Their Cholestasis-Related Targets

**Figure 2:**
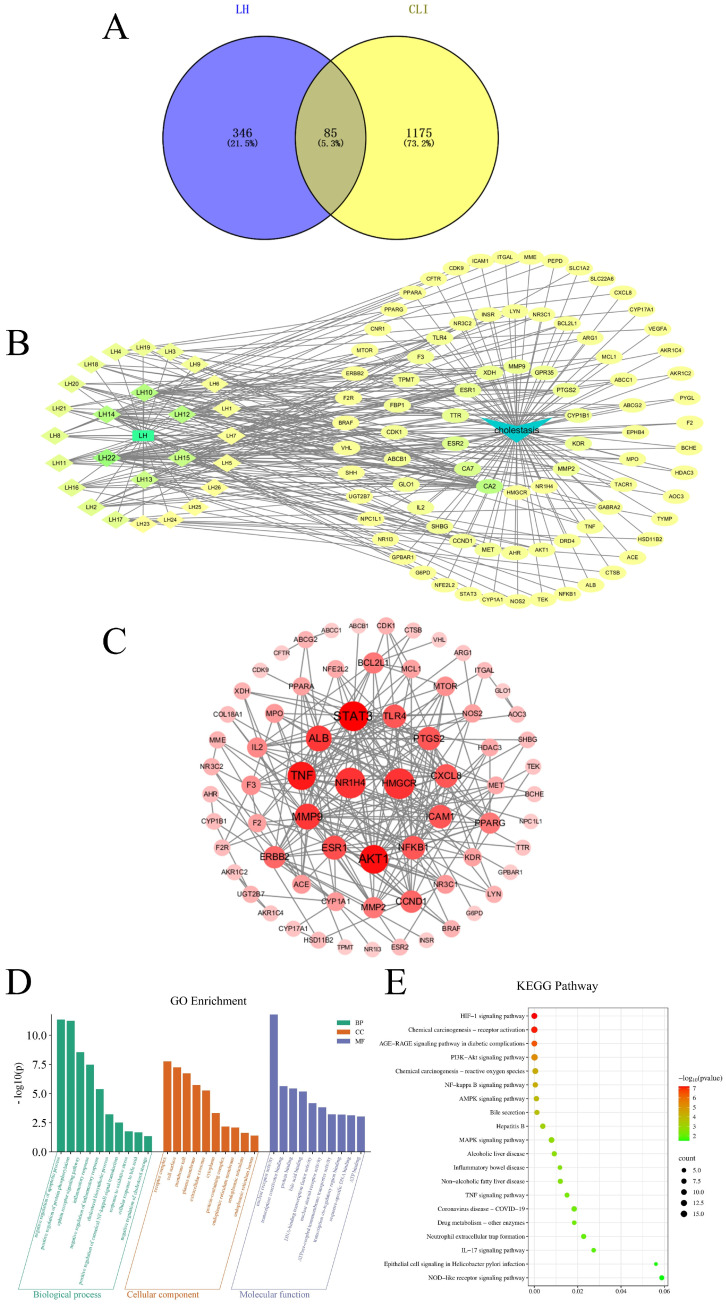
Network pharmacology findings for LH in cholestasis therapy. (**A**) Venn diagram of LH and cholestasis-related targets. (**B**) Drug–Disease–Ingredient–Target (DDIT) network (the V means cholestasis; the round rectangle means LH; the ellipses mean the intersectional targets; the diamonds represent the serum detected chemical components in rats). (**C**) The PPI network constructed from intersecting core targets (the larger node sizes and darker node colours indicate greater importance of the corresponding genes). (**D**) GO enrichment analysis histogram. MF = molecular fictions, CC = cell composition, BP = biological processes. (**E**) KEGG enrichment bubble plot. Bubble color represents the adjusted *p*-value, and bubble size represents the number of enriched genes.

In total, 26 serum-absorbed constituents were detected in rats (Table 2), and their corresponding gene targets were retrieved from the Swiss Target Prediction database. Furthermore, 1175 cholestasis targets were retrieved from three disease databases. By analyzing the overlapping genes between constituents and cholestasis, 85 potential therapeutic targets were obtained (Figure 2A).

**Table 2:** Identification of serum-absorbed constituents in rats after oral administration of LH.

No.	Compound	Molecular Formula	*m*/*z*	RT (min)	ppm	Fragment Ion	Adduct
1	Valine	C_5_H_11_NO_2_	118.0863	1.002	0.03	72.0802, 59.0726	[M + H]^+^
2	p-coumaric acid	C_9_H_8_O_3_	165.0547	1.269	0.61	147.0432, 119.0492	[M + H]^+^
3	Eugenol	C_10_H_12_O_2_	165.0910	1.338	3.64	79.0552, 109.0502	[M + H]^+^
4	2-Hydroxycinnamic acid	C_9_H_8_O_3_	165.0547	1.339	0.61	65.0383, 91.0545	[M + H]^+^
5	Isoleucine	C_6_H_13_NO_2_	132.1017	1.369	−1.51	86.0963, 69.0699	[M + H]^+^
6	Phenylalanine	C_9_H_11_NO_2_	166.0864	2.153	−1.81	120.0808, 103.0539, 79.0536, 91.0535	[M + H]^+^
7	Catechol	C_6_H_6_O_2_	109.0293	3.277	1.84	108.0219, 65.0038, 91.0189	[M − H]^−^
8	Tryptophan	C_11_H_12_N_2_O_2_	203.0826	3.759	0.06	116.0516, 65.0146	[M − H]^−^
9	2,6-Dimethoxyphenol	C_8_H_10_O_3_	155.0704	7.973	3.22	91.0531, 99.0434	[M + H]^+^
10	Genistein	C_15_H_10_O_5_	271.0609	8.856	2.95	107.0867, 197.8706	[M + H]^+^
11	Gallic acid	C_7_H_6_O_5_	169.0155	8.889	4.24	125.0236, 107.0087	[M − H]^−^
12	10-gingerol	C_21_H_34_O_4_	351.2509	10.600	−4.56	137.0235	[M + H]^+^
13	Epicatechin	C_15_H_14_O_6_	291.0845	10.861	−5.15	111.0481	[M + H]^+^
14	L-Menthol	C_10_H_20_O	174.1852	11.224	0.02	55.0542, 57.0699, 93.0699	[M + NH_4_]^+^
15	Ferulic acid	C_10_H_10_O_4_	217.0468	13.626	−1.38	128.0632, 177.1049	[M + Na]^+^
16	Umbelliferone	C_9_H_6_O_3_	163.0392	13.730	−0.05	77.0386, 92.0256, 51.0228	[M + H]^+^
17	Protocatechuic acid	C_7_H_6_O_4_	153.0206	14.998	4.60	108.0216, 65.0032, 109.0295	[M − H]^−^
18	Nardosinone	C_15_H_22_O_3_	251.1639	15.852	−0.80	67.0542, 79.0542, 81.0699	[M + H]^+^
19	Syringic acid	C_9_H_10_O_5_	197.0459	17.179	1.78	153.0296, 182.0018	[M − H]^−^
20	Undulatoside A	C_16_H_18_O_9_	355.1033	18.065	2.25	175.1412, 193.1232	[M + H]^+^
21	Dihydroxy-methoxy-methyl homoisoflavanone	C_18_H_18_O_5_	337.1047	20.553	1.19	209.1649, 69.069, 70.0735	[M + Na]^+^
22	Myricitrin	C_21_H_20_O_12_	465.1028	22.211	0.86	319.9724	[M + H]^+^
23	Dimethylophiopogonanone	C_20_H_22_O_5_	365.1356	22.627	−0.65	91.0531, 119.0855	[M + Na]^+^
24	2-Methoxycinnamaldehyde	C_10_H_10_O_2_	163.0754	22.730	−0.11	77.0386, 51.0229	[M + H]^+^
25	Kaempferide	C_16_H_12_O_6_	301.0707	23.305	1.99	161.9449, 229.9486	[M + H]^+^
26	Isoschaftoside	C_26_H_28_O_14_	565.1571	29.732	3.36	389.0598, 353.1024, 273.0406	[M + H]^+^

The topological structure of the DDIT network was analyzed and visualized using Cytoscape (Figure 2B). The Network Analyzer tool was used to compute network degree values, where a higher degree signifies a stronger relationship between a component and its target. The constructed network comprised 113 nodes, which included one drug node, one disease node, 26 ingredient nodes, and 85 target nodes. The top six components were identified as core candidate constituents (Table 3).

**Table 3:** Core serum-absorbed constituents identified by network topology analysis.

No.	Components
1	Kaempferide (LH22)
2	Ferulic acid (LH14)
3	Genistein (LH10)
4	10-gingerol (LH12)
5	Umbelliferone (LH13)
6	L-Menthol (LH15)

### 3.3. PPI Network Analysis

Following submission of the 85 intersecting genes to the STRING database, only targets with a confidence score greater than 0.7 were chosen and later transferred to Cytoscape for network generation. The resulting network was then analyzed using the Network Analyzer tool to calculate the average node degree and establish the PPI network of LH targets related to cholestasis. In the constructed PPI network, larger node sizes and darker node colors indicate greater importance of the corresponding genes (Figure 2C). Thirteen core targets, including AKT1, STAT3, TNF, HMGCR, and NR1H4, were identified through screening.

### 3.4. KEGG Pathway and GO Enrichment Analysis

To clarify the mechanisms underlying the effects of LH on cholestasis, KEGG pathway and GO enrichment analysis were conducted. Figure 2D,E presents the top 10 terms from the three GO categories-biological process (BP), cellular component (CC), and molecular function (MF)-as well as 20 significantly enriched KEGG signaling pathways. This result indicated that the acquired targets were enriched in inflammatory response, cholesterol biosynthetic process, cellular response to bile acids, response to oxidative stress, and negative regulation of cholesterol storage, which are closely related to the pathological mechanisms of cholestasis.

Furthermore, KEGG pathway enrichment analysis demonstrated that the 85 potential targets were involved in 20 KEGG pathways, which were further classified into several categories: (1) Pathways directly governing bile secretion, including the Bile secretion pathway; (2) Signal transduction pathways related to oxidative stress and inflammation, including IL-17 signaling pathway, NF-κB signaling pathway; (3) Pathways related to cellular metabolism and stress, for example, AMPK signaling pathway; (4) Others.

### 3.5. Molecular Docking

**Figure 3:**
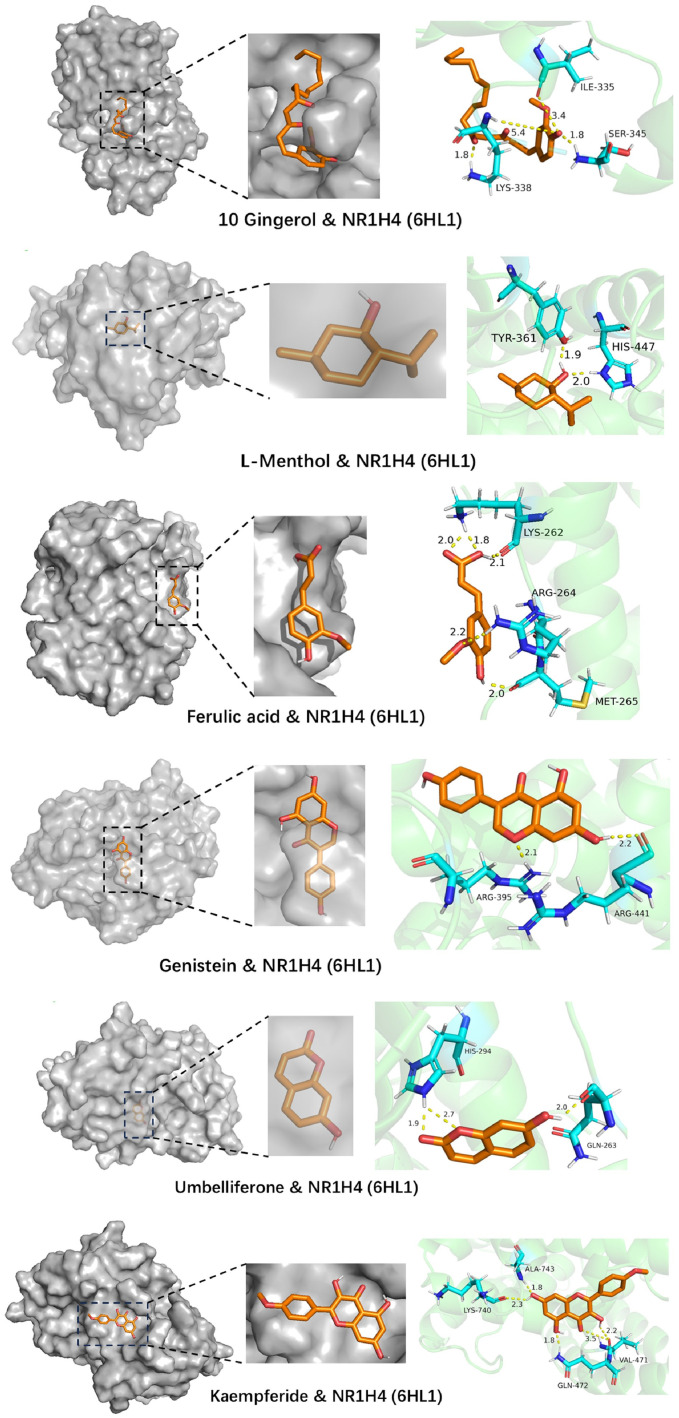
Molecular docking analysis for validating interactions between candidate compounds and key target (FXR). Orange sticks represent the candidate ligands, and blue/cyan sticks represent the interacting amino acid residues.

Based on the network pharmacology results, 6 core bioactive components were selected as representative ligands for molecular docking with the target protein NR1H4 (FXR), respectively. In such docking simulations, the binding of an active ingredient to its target protein brings the ligand’s functional groups into close contact with the protein’s active site. This interaction may induce conformational changes and functional alterations in the protein, consequently influencing associated signaling pathways. The compound acts as a ligand, whereas the protein functions as the receptor. In general, a lower binding energy between a receptor and its ligand indicates a more stable interaction. According to established criteria, binding energies below −4.25 kcal/mol (1 kcal ≈ 4.18585 kJ) suggest a certain level of binding activity. A threshold of −5.0 kcal/mol is considered indicative of good binding activity, while values lower than −7.0 kcal/mol reflect high binding activity [23,24]. Applying these criteria, our docking analysis demonstrated that the active ingredients possessed high binding affinities with FXR (Table 4). These results suggest that the candidate constituents may interact with FXR. The resulting docking conformations were subsequently visualized using PyMOL (Figure 3).

### 3.6. Effects of LH on Liver Function

**Figure 4:**
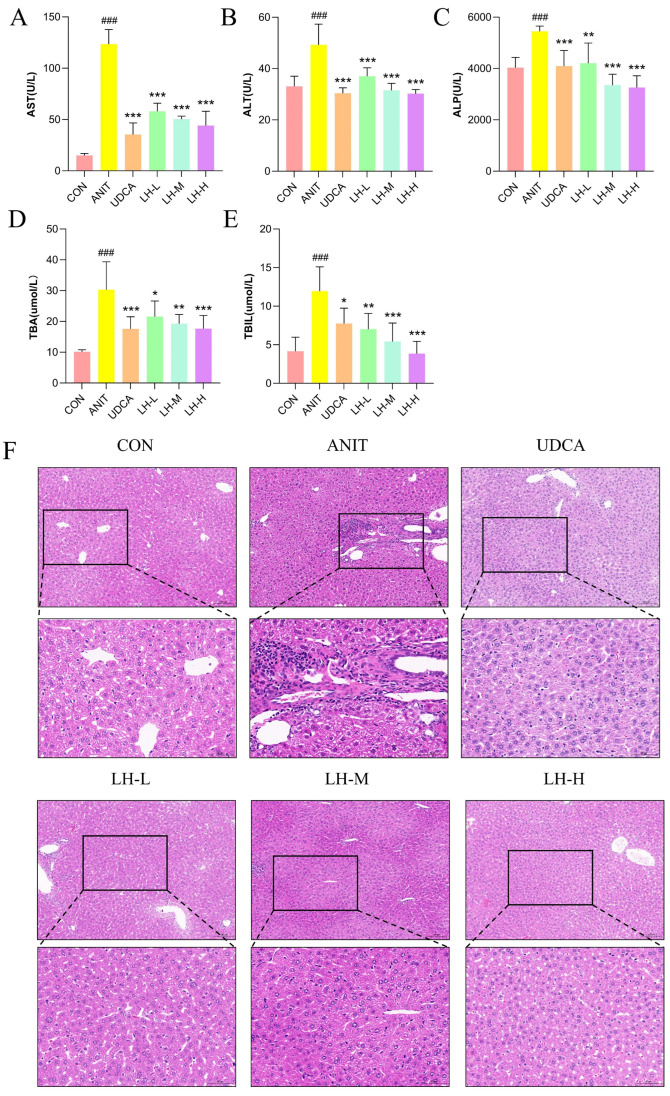
LH administration effectively attenuated ANIT-induced liver injury. Panels (**A**–**E**) show the serum levels of liver function biomarkers, including ALP, ALT, AST, TBA, and TBIL. Panel (**F**) presents H&E-stained liver sections, with scale bars of 100 μm and 50 μm. All data are expressed as mean ± SEM (*n* = 8). Statistical significance is indicated as ^###^ *p* < 0.001 versus the Control group, and * *p* < 0.05, ** *p* < 0.01, *** *p* < 0.001 versus the ANIT group.

To assess the effects of LH against CLI, serum concentrations of indicators including ALP, AST, ALT, TBA, and TBIL were measured. As depicted in Figure 4, ANIT treatment caused a marked increase in these markers, confirming the successful induction of CLI. Treatment with LH at varying doses provided potent protection against ANIT-induced hepatic injury, as evidenced by dose-dependent reductions in serum levels of ALP, ALT, AST, TBA, and TBIL (Figure 4A–E). These decreases were statistically significant (*p* < 0.05, *p* < 0.01, *p* < 0.001) relative to the ANIT group.

H&E staining demonstrated that, whereas control hepatocytes retained normal morphology and lacked focal necrosis, those in the ANIT group were characterized by swelling, degeneration, necrosis, dilated hepatic sinusoids, and inflammatory cell infiltration (Figure 4F). Histological examination revealed that LH administration markedly ameliorated ANIT-induced liver injury. Notably, the high-dose LH treatment substantially attenuated hepatic parenchymal necrosis and inflammatory infiltration, while also improving cholestasis within the intrahepatic bile ducts.

### 3.7. RNA-Seq Analysis

To gain deeper insight into how LH modulates BA metabolism and alleviates cholestasis, we performed a transcriptome analysis to detect possible targets and pathways associated with LH-mediated regulation of BA metabolism. Serum biochemistry and pathological analyses demonstrated that the LH-H group exhibited superior amelioration of CLI compared to the other LH-treated groups. Accordingly, transcriptome sequencing was performed on samples from control, ANIT, and LH-H groups.

**Figure 5:**
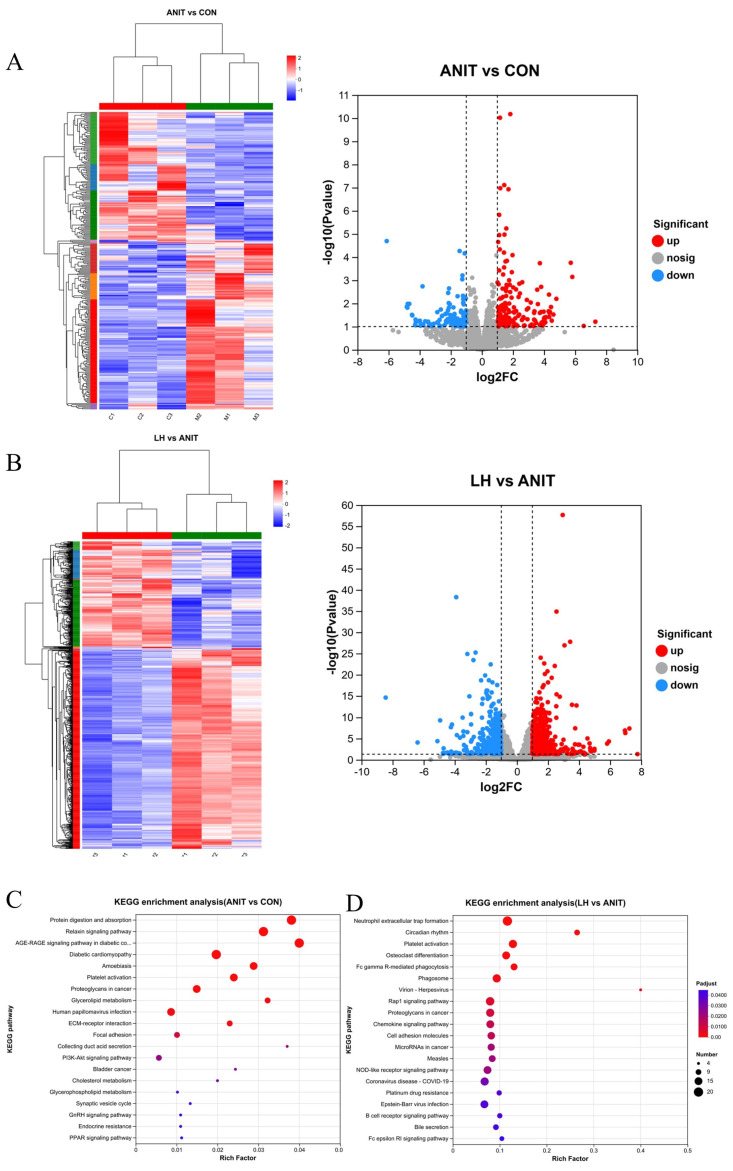
Differential gene expression analysis of the mRNA-Seq. (**A**) Comparison of gene expression profiles between the Control group and the ANIT group. (**B**) Comparison of gene expression profiles between the ANIT group and the LH treatment group. (**C**,**D**) KEGG enrichment analysis.

As confirmed by library quality assessment, the constructed libraries were of high quality and reliability, which ensured their suitability for subsequent bioinformatics analyses. In comparison with the control group, 284 genes were differentially expressed in the ANIT group, consisting of 162 upregulated and 122 downregulated genes (Figure 5A). Conversely, relative to the ANIT group, 973 genes exhibited significant alterations in the LH treatment group, with 638 upregulated and 335 downregulated (Figure 5B). These results indicated that LH treatment markedly reshaped the transcriptomic profile altered by ANIT, suggesting that these differentially expressed genes could serve as regulatory targets of LH in the treatment of cholestasis.

To elucidate the mechanism of action of LH in cholestasis, we focused on the target genes implicated in its therapeutic effect. KEGG enrichment analysis (Figure 5C,D) identified multiple liver disease-related pathways, including those for bile secretion, alcoholic liver injury, and inflammation. Notably, the bile secretion pathway, which is directly relevant to cholestasis, ranked among the top 20 enriched pathways. This result supports the premise that LH alleviates cholestasis in the ANIT-induced CLI model, at least in part, by modulating this critical pathway.

### 3.8. Effects of LH on FXR-Related Bile Acid Homeostasis Markers In Vivo

**Figure 6:**
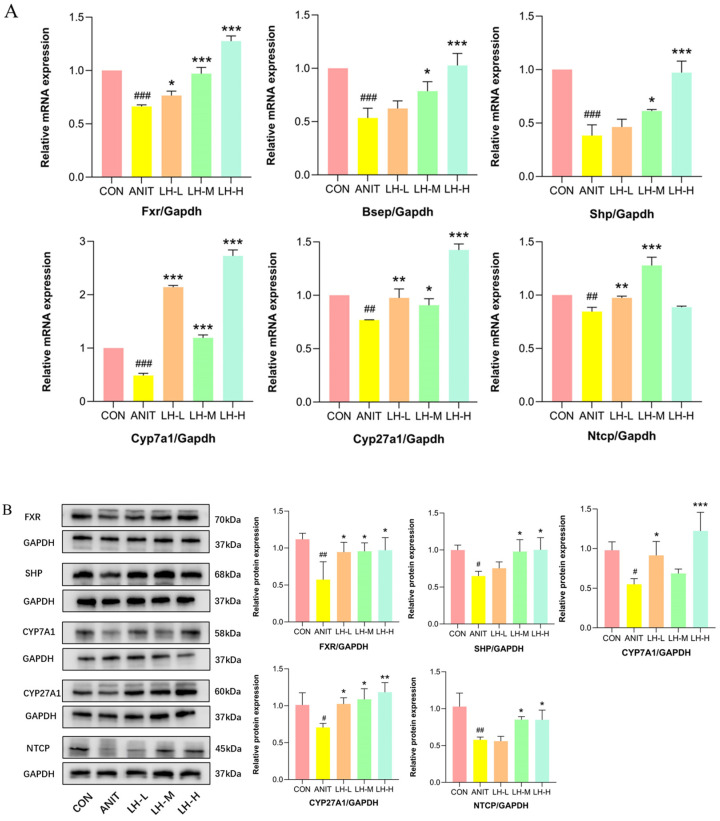
Effects of LH on FXR-related bile acid homeostasis markers in liver tissue. (**A**) The mRNA expression levels of Cyp27a1, Bsep, Fxr, Shp, Cyp7a1, and Ntcp, normalized to Gapdh, relative to the control group. (**B**) Western blot analysis of target proteins (CYP7A1, SHP, FXR, CYP27A1, and NTCP). Expression normalized to GAPDH (internal control) and presented relative to the control group. Statistics were mean ± SEM (*n* = 3; ^#^ *p* < 0.05, ^##^ *p* < 0.01, ^###^ *p* < 0.001, compared to the Control group * *p* < 0.05, ** *p* < 0.01, *** *p* < 0.001, compared with the ANIT group).

The effects of LH on key genes involved in bile acid synthesis, transport, and FXR signaling were examined in mouse liver tissues. qPCR analysis (Figure 6A) revealed that ANIT treatment significantly reduced the mRNA levels of Cyp27a1, Bsep, Fxr, Shp, Cyp7a1, and Ntcp compared with the control group. Importantly, LH administration effectively restored the expression of these genes, indicating a reversal of ANIT-induced transcriptional suppression.

Consistent with the mRNA changes, Western blotting (Figure 6B) showed that protein levels of CYP7A1, SHP, FXR, CYP27A1, and NTCP were markedly lower in the ANIT group than in controls. LH treatment substantially reversed these decreases, bringing protein expression back toward normal levels. Given that these genes and proteins are critical regulators of BA synthesis, efflux, uptake, and FXR-mediated signaling, these findings are consistent with the interpretation that LH alleviates cholestatic liver injury by restoring BA homeostasis in association with FXR pathway regulation.

### 3.9. Effects of LH on FXR Pathway-Related Protein Expression In Vitro

**Figure 7:**
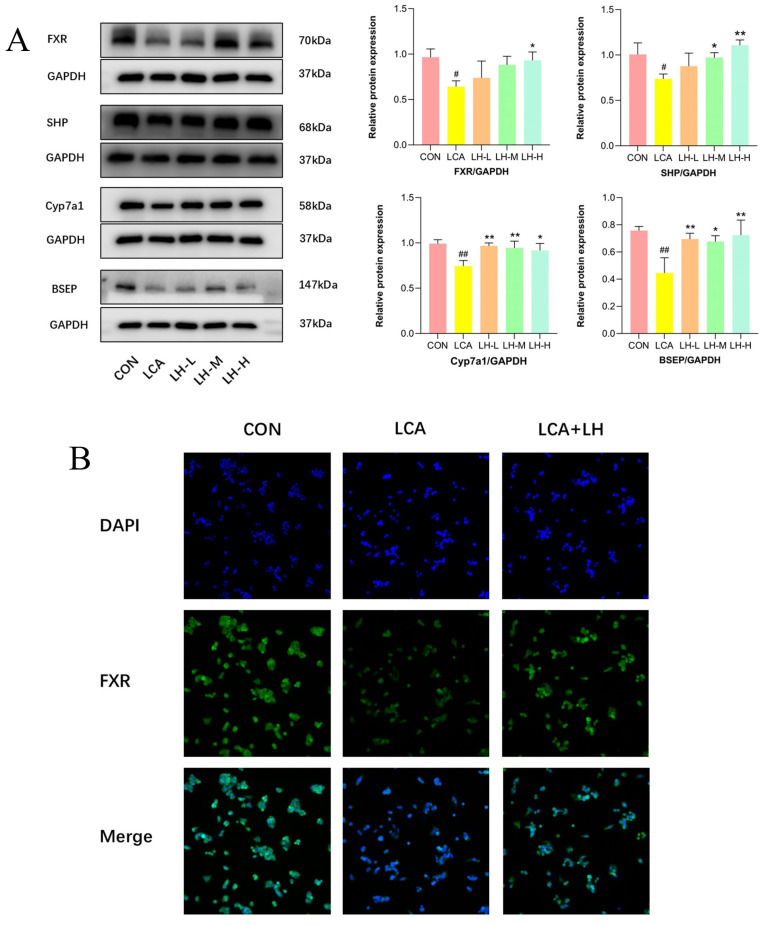
Effects of LH on bile secretion-related protein expression in HepG2 cells. (**A**) Representative Western blot bands and quantification of target proteins (FXR, SHP, CYP7A1, BSEP). All protein levels were normalized to GAPDH as an internal loading control and are expressed relative to the control group. (**B**) Immunofluorescence staining showing FXR protein expression and localization. Data are presented as mean ± SEM (*n* = 3). Statistical significance: ^#^ *p* < 0.05, ^##^ *p* < 0.01 versus the control group; * *p* < 0.05, ** *p* < 0.01, versus the LCA group.

To further validate the involvement of the FXR pathway, HepG_2_ cells were treated with lithocholic acid (LCA), a known FXR inhibitor and cholestasis inducer. Western blot analysis (Figure 7A) demonstrated that LCA treatment significantly downregulated the protein expression of BSEP, SHP, FXR, and CYP7A1 compared with the control group. Conversely, co-treatment with LH dose-dependently restored the levels of these proteins, suggesting that LH counteracts LCA-induced suppression of the FXR signaling cascade.

Immunofluorescence staining (Figure 7B) was used to assess FXR expression and localization in HepG_2_ cells. In line with the Western blot results, FXR fluorescence intensity was markedly reduced in the LCA-treated group, whereas LH treatment significantly upregulated FXR expression. Together, these in vitro findings corroborate the in vivo observations and support the involvement of the FXR signaling pathway in the protective effects of LH against cholestatic liver injury.

## 4. Discussion

CLI remains a challenging condition with limited therapeutic options. In this study, we employed an integrated strategy combining serum pharmacochemistry, network pharmacology, transcriptomics, and experimental validation to systematically investigate the chemical composition, in vivo absorbed constituents, and molecular mechanisms of LH in CLI. Our findings provide convergent evidence that LH alleviates CLI, at least partly in association with FXR-related bile acid homeostatic regulation.

Using UPLC-Q-TOF-MS/MS, a total of 129 chemical constituents were identified in LH, among which 26 components were detected in rat serum after oral administration. These absorbed constituents, including phenolic acids, flavonoids, terpenoids, phenylpropanoids, and amino acids, are considered the primary bioactive forms responsible for the in vivo effects of LH. Notably, several of these compounds have been independently reported to possess hepatoprotective properties. For instance, protocatechuic acid ameliorates hepatic steatosis by modulating lipid metabolism and gut microbiota [25]; gallic acid elicits antioxidant and anti-inflammatory responses by neutralizing free radicals, reducing malondialdehyde formation, and lowering pro-inflammatory marker levels [26]; and epicatechin alleviates liver oxidative and inflammatory injury [27]. Collectively, these results support the notion that the multi-component nature of LH contributes to its hepatoprotective activity.

To gain insight into the molecular targets and pathways mediating the anti-cholestatic effects of LH, network pharmacology and PPI network analyses were performed. From the 26 absorbed components, 85 targets overlapping with cholestasis-related targets were identified, among which AKT1, STAT3, TNF, HMGCR, and NR1H4 (FXR) emerged as core targets. Notably, FXR is a master regulator of BA synthesis and metabolism and represents a well-established therapeutic target in cholestasis [28,29,30]. GO and KEGG enrichment analyses further indicated that the therapeutic action of LH involves biological processes such as inflammatory response, cholesterol biosynthesis, and cellular response to BA, with the bile secretion pathway being the most significantly enriched pathway. These predictions were further corroborated by molecular docking, which demonstrated favorable binding affinities between the top six active components of LH and FXR, suggesting that these constituents may directly modulate FXR activity [31].

The in vivo protective effects of LH were evaluated in an ANIT-induced CLI mouse model. ANIT is a well-established agent that induces intrahepatic cholestasis by triggering inflammatory injury to small intrahepatic bile ducts, leading to hepatocyte damage [32]. The pathological features of ANIT-induced hepatotoxicity closely mimic drug-induced hepatitis in humans, making this model highly relevant for studying intrahepatic cholestasis [33]. In the present study, ANIT administration resulted in marked elevations of serum ALT, ALP, AST, TBA, and TBIL, biomarkers commonly used to assess CLI, along with extensive hepatocellular necrosis and inflammatory infiltration [34,35]. These changes were dose-dependently reversed by LH treatment, demonstrating that LH effectively ameliorates ANIT-induced liver injury and cholestasis.

RNA-sequencing analysis of liver tissues from control, ANIT-treated, and LH-treated mice revealed that LH significantly reversed the expression of genes associated with BA metabolism and FXR signaling. Among the enriched pathways, the bile secretion pathway ranked among the top 20, further supporting the involvement of this pathway in the therapeutic action of LH. Subsequent qPCR and Western blot analyses confirmed that ANIT treatment significantly suppressed the mRNA and protein levels of FXR, SHP, CYP7A1, CYP27A1, BSEP, and NTCP, whereas LH treatment effectively restored their expression. As a rate-limiting enzyme in the classical BA synthesis pathway, CYP7A1 is tightly regulated by FXR [36]. In cholestasis, excessive intrahepatic BA accumulation suppresses CYP7A1 activity [37], and the restoration of CYP7A1 expression by LH suggests that LH contributes to the re-establishment of BA metabolic homeostasis.

To further test whether the effects of LH are mediated through the FXR pathway, we employed an LCA-induced cellular model of cholestasis. LCA is a known FXR inhibitor and inducer of intrahepatic cholestasis, making it suitable for dissecting the mechanistic involvement of FXR [38,39]. In LCA-treated HepG_2_ cells, FXR, SHP, CYP7A1, and BSEP protein levels were significantly downregulated, whereas LH treatment dose-dependently restored their expression. Immunofluorescence staining confirmed that FXR expression was markedly reduced in LCA-treated cells and upregulated by LH. These in vitro findings align with the in vivo observations and collectively indicate that LH alleviates CLI at least in part through activation of the FXR signaling pathway.

The nuclear receptor FXR functions as an endogenous BA sensor and maintains BA homeostasis by inhibiting BA synthesis and promoting BA transport. Under physiological conditions, activation of the hepatic FXR/SHP pathway leads to repression of CYP7A1 and CYP27A1, thereby suppressing BA synthesis by both the classical and alternative pathways [40,41]. In addition, FXR activation reduces BA uptake by downregulating NTCP and enhances BA efflux by upregulating BSEP [7]. In the present study, LH treatment restored FXR, SHP, and BSEP expression, supporting the involvement of FXR-related BA regulatory signaling. Notably, LH also restored NTCP expression in ANIT-treated mice. NTCP is a major hepatic BA uptake transporter [42], and the restoration of NTCP expression by LH may promote hepatic reuptake of peripheral BAs, thereby facilitating enterohepatic circulation and contributing to the resolution of cholestasis [43]. Similarly, although FXR activation is generally associated with CYP7A1 repression, we observed that LH increased both FXR/SHP and CYP7A1/CYP27A1 expression. This apparent discrepancy is not unique to our study and has also been observed in other CLI models [44,45].

One possible explanation is that the expression pattern observed in this study reflects the recovery of a disturbed BA homeostatic network rather than the direct response to a single FXR agonist under physiological conditions. CYP7A1 regulation is highly context-dependent and is not governed solely by the hepatic FXR/SHP axis [46,47]. In addition to hepatic FXR/SHP-mediated repression, CYP7A1 expression can be influenced by the intestinal FXR–FGF15/19–FGFR4 feedback pathway, LRH-1/HNF4α-dependent transcriptional regulation, BA composition, and inflammatory signaling [47]. Moreover, cholestasis is associated with complex transcriptional and post-transcriptional alterations in hepatobiliary transporters and metabolic enzymes, while inflammatory cytokines can suppress hepatic organic anion transporters, including Ntcp, during toxic liver injury and cholestasis [48,49]. Therefore, the restoration of CYP7A1, CYP27A1, and NTCP expression after LH treatment may reflect reduced toxic BA burden, attenuation of inflammatory stress, and recovery of hepatocyte function rather than a contradiction of FXR-related regulation. Taken together, these findings suggest that LH regulates an integrated BA homeostatic network rather than acting as a simple FXR agonist.

Several limitations of this study should be acknowledged. First, although network pharmacology, molecular docking, transcriptomic analysis, and both in vivo and in vitro validation consistently implicated FXR-related bile acid regulation in the protective effects of LH, the current evidence remains largely convergent and associative. The present study demonstrated coordinated changes in FXR and several bile acid homeostasis-related targets, including SHP, CYP7A1, CYP27A1, BSEP, and NTCP, but did not include direct FXR loss-of-function experiments. Therefore, FXR should be interpreted as a strongly implicated pathway rather than a definitively proven indispensable target of LH. Future studies using FXR-specific antagonists, siRNA- or CRISPR-mediated FXR silencing, and FXR-deficient animal models are required to establish the causal contribution of FXR signaling. Second, although 26 blood-absorbed components of LH were identified by serum pharmacochemistry, the specific compound(s) responsible for FXR-related regulation and the quantitative contribution of each component remain to be determined. Further validation using purified serum-absorbed constituents is needed to clarify the active material basis of LH. Third, the ANIT-induced mouse model and LCA-treated HepG_2_ cell model cannot fully recapitulate the diverse pathological spectrum of human cholestatic liver diseases. Therefore, additional models representing different forms of cholestasis, such as primary biliary cholangitis, primary sclerosing cholangitis, drug-induced cholestasis, and obstructive cholestasis, will be required to further confirm the generalizability of our findings. In addition, the potential contributions of other signaling pathways, such as inflammatory and oxidative stress-related pathways, cannot be excluded and merit further investigation.

## 5. Conclusions

In this study, an integrated strategy combining chemical profiling, serum pharmacochemistry, network pharmacology, transcriptomic analysis, and experimental validation was used to investigate the potential material basis and anti-cholestatic mechanisms of LH. UPLC-Q-TOF-MS/MS analysis identified 129 chemical constituents in LH, and serum pharmacochemistry detected 26 blood-absorbed components, which may represent the potential bioavailable chemical basis of LH in vivo. Network pharmacology, molecular docking, and transcriptomic analysis suggested that bile acid metabolism and FXR-related signaling may be involved in the protective effects of LH against CLI. In vivo and in vitro experiments further showed that LH alleviated cholestatic liver injury and regulated several FXR-related bile acid homeostasis markers, including SHP, CYP7A1, CYP27A1, BSEP, and NTCP. Collectively, these findings suggest that LH may protect against CLI, at least in part, by modulating FXR-associated bile acid homeostatic regulation. These results provide experimental evidence for further pharmacological investigation and potential development of LH as a therapeutic candidate for cholestatic liver injury.

## Figures and Tables

**Table 1:** Primer sequences.

Gene Name	Primer	Mouse Sequence (5′–3′)
Shp	Forward	GCACGATCCTCTTCAACCCA
	Reverse	CAGAAGGGTGCCTGGAATGT
Cyp7a1	Forward	CTGGGGGATTGCTGTGGTAG
	Reverse	CCAGGTATGGAATCAACCCGT
Cyp27a1	Forward	TGATGCTGTCAAGGCTGGTA
	Reverse	TCACCTTCTTGCTGGGAACC
Fxr	Forward	GCTTGATGTGCTACAAAAGCTG
	Reverse	CGTGGTGATGGTTGAATGTCC
Ntcp	Forward	CCTTGCGCCATAGGGATCTT
	Reverse	AGGCATCAGGGAGGAGGTAG
Bsep	Forward	TCTGACTCAGTGATTCTTCGCA
	Reverse	CCCATAAACATCAGCCAGTTGT
Gapdh	Forward	TGTGTCCGTCGTGGATCTGA
	Reverse	TTGCTGTTGAAGTCGCAGGAG

**Table 4:** Molecular docking results of major candidate constituents with FXR.

Target	Component	Maximum Binding Energy/(kcal/mol)
FXR	L-Menthol	−5.78
FXR	10-Gingerol	−6.45
FXR	Genistein	−6.09
FXR	Ferulic acid	−4.98
FXR	Umbelliferone	−5.91
FXR	Kaempferide	−5.13

## Data Availability

The original contributions presented in the study are included in the article/Supplementary Materials; further inquiries can be directed to the corresponding author.

## Supplementary Materials

The following supporting information can be downloaded at: https://www.mdpi.com/article/10.3390/cimb48070682/s1.

## Abbreviations

The following abbreviations are used in this manuscript:

LH	Lysimachiae Herba
CLI	cholestatic liver injury
UPLC-Q-TOF-MS/MS	ultra-performance liquid chromatography-quadrupole-time-of-flight mass spectrometry
UDCA	ursodeoxycholic acid
BA	bile acid
ANIT	1-naphthyl isothiocyanate
LCA	lithocholic acid
ALP	alkaline phosphatase
ALT	alanine aminotransferase
TBA	total bile acid
AST	aspartate aminotransferase
TBIL	total bilirubin
FXR	farnesoid X receptor
CYP7A1	cholesterol 7α-hydroxylase
NTCP	sodium-taurocholate cotransporting polypeptide
BSEP	bile salt export pump
SHP	small heterodimer partner
CYP27A1	cytochrome P450 family 27 subfamily A member 1
HMGCR	3-hydroxy-3-methylglutaryl-CoA reductase
GAPDH	glyceraldehyde-3-phosphate dehydrogenase
H&E	hematoxylin-eosin
DDIT	drug–disease–ingredient–target
PPI	protein–protein interaction
GO	Gene Ontology
KEGG	Kyoto Encyclopedia of Genes and Genomes
RNA-seq	RNA-sequencing
qRT-PCR	quantitative real-time polymerase chain reaction
PBC	primary biliary cholangitis
PSC	primary sclerosing cholangitis
SPF	specific-pathogen-free
BCA	bicinchoninic acid
PVDF	polyvinylidene fluoride
BSA	bovine serum albumin
PBS	phosphate-buffered saline
DEGs	differentially expressed genes
