# Supplementary material for: Lysimachiae Herba Modulates FXR to Alleviate Cholestatic Liver Injury: Insights from Serum Pharmacochemistry and Experimental Validation
Source: Curr Issues Mol Biol. 2026 Jul 2;48(7):682. doi: 10.3390/cimb48070682 (PMC13407394; doi:10.3390/cimb48070682)
Supplement: Supplementary file 1 [file cimb-48-00682-s001.zip › cimb-4309546-supplementary.pdf]

Table S1

Identification results of chemical constituents of LH.

No.	Compound	Molecular Formula	*m/z*	RT (min)	ppm	Fragment Ion	Adduct
1	Ethyl cinnamate	$C_{11}H_{12}O_2$	194.1176	0.396	0.39	107.0496、77.0384、79.0544	$[M+NH_4]+$
2	Adenine	$C_5H_5N_5$	136.0616	0.673	-1.47	118.9210、56.9643、55.0296	[M+H] +
3	Proline	$C_5H_9NO_2$	114.0567	0.857	5.26	74.0254、66.9971	[M-H]-
4	D-Mannonic acid	$C_6H_{12}O_7$	195.0512	0.909	-1.03	75.0088、129.0195、59.0138、177.0406	[M-H]-
5	Valine	$C_5H_{11}NO_2$	118.0863	0.958	0.61	72.0801、59.0725	[M+H] +
6	Ribose	$C_5H_{10}O_5$	149.0455	0.995	1.44	55.0189、71.0139、73.0295	[M-H]-
7	Citric acid	$C_6H_8O_7$	191.0199	1.082	0.9	57.0346、67.0188、87.0087	[M-H]-
8	Malic acid	$C_4H_6O_5$	133.0140	1.255	-1.80	71.0139、115.0037	[M-H]-
9	p-coumaric acid	$C_9H_8O_3$	165.0547	1.269	0.68	147.0432、119.0492	[M+H] +
10	Eugenol	$C_{10}H_{12}O_2$	165.0910	1.338	3.64	79.0552、109.0502	[M+H] +
11	2-Hydroxycinnamic acid	$C_9H_8O_3$	165.0547	1.339	0.68	65.0383、91.0545	[M+H] +
12	Isoleucine	$C_6H_{13}NO_2$	132.1020	1.369	0.76	86.0964、69.0699	[M+H] +
13	Protocatechuic aldehyde	$C_7H_6O_3$	183.0299	1.687	0.06	124.0163、78.0103、108.0222	[M+HCOO]-
14	7-Methylcoumarin	$C_{10}H_8O_2$	178.0863	1.978	0.10	162.0548、89.0384、91.0544	$[M+NH_4]+$
15	Phenylalanine	$C_9H_{11}NO_2$	166.0864	2.154	0.60	120.0807、103.0540、79.0536、91.0534	[M+H] +

16	2,5-Dihydroxybenzoic acid	$C_7H_6O_4$	153.0193	2.239	-0.10	78.9586、68.9955、65.0033、79.0189	[M-H]-
17	Leonuriside A	$C_{14}H_{20}O_9$	355.1000	2.609	-0.45	192.0417、178.0108	[M+Na] +
18	Anisaldehyde	$C_8H_8O_2$	135.0449	2.655	-1.49	89.0397、65.0033	[M-H]-
19	Epigallocatechin	$C_{15}H_{14}O_7$	305.0666	2.914	-0.29	125.0239、167.0354、179.0333、137.0244	[M-H]-
20	5,7,3', -Trihydroxy-6,4',5,-trimethoxyflavone	$C_{18}H_{16}O_8$	383.0737	3.067	-1.85	207.0204、145.0268、134.0962	[M+Na] +
21	Maltol	$C_6H_6O_3$	127.0389	3.274	-0.79	55.9342、71.9283、51.0227	[M+H] +
22	Catechol	$C_6H_6O_2$	109.0293	3.277	0.28	108.0220、65.0038、91.0190	[M-H]-
23	Dihydromyricetin	$C_{15}H_{12}O_8$	321.0594	3.412	-4.98	165.0551、139.0394	[M+H] +
24	Phenyl benzoate	$C_{13}H_{10}O_2$	216.1018	3.481	-0.53	77.0383、56.9641、91.0558	[M+NH4] +
25	Sorbic acid	$C_6H_8O_2$	113.0596	3.550	-0.88	67.0543、55.0537	[M+H] +
26	Chlorogenic acid	$C_{16}H_{18}O_9$	353.0868	3.622	-2.70	191.0558、179.0349、135.0445、85.0285	[M-H]-
27	Tryptophan	$C_{11}H_{12}N_2O_2$	203.0826	3.760	0.06	116.0516、65.0148	[M-H]-
28	Salidroside	$C_{14}H_{20}O_7$	323.1103	3.895	0.42	83.0603、57.0329、122.0722	[M+Na] +
29	p-Hydroxybenzoic acid	$C_7H_6O_3$	137.0244	4.159	0.09	108.0214、118.9409、65.9985、93.0295	[M-H]-
30	2-Amino-3-(1H-indol-2-yl)propanoic acid	$C_{11}H_{12}N_2O_2$	205.0973	4.172	0.66	118.065、91.0543、89.0384	[M+H] +

31	Higenamine	$C_{16}H_{17}NO_3$	272.3208	4.397	-0.25	107.0491、115.0542、77.00386	[M+H] +
32	Methyl gallate	$C_8H_8O_5$	183.0298	4.591	-0.61	78.0113、124.0167、67.0181	[M-H]-
33	3-(4-hydroxyphenyl)propanoic acid	$C_9H_{10}O_3$	165.0558	4.643	0.51	150.0322、122.9701、59.0134	[M-H]-
34	Gentiopicrin	$C_{16}H_{20}O_9$	379.1000	4.776	0.30	172.9891、209.0074	[M+Na] +
35	Sucrose	$C_{12}H_{22}O_{11}$	341.1087	4.851	0.59	179.0345、89.0245、59.0132	[M-H]-
36	Kaempferol-3-sophoroside-7-glucoside	$C_{33}H_{40}O_{21}$	795.1937	4.862	-2.14	347.0941、633.1430、323.9894	[M+Na] +
37	p-Coumaric acid 4-O-β-D-glucopyranside	$C_{15}H_{18}O_8$	349.0893	4.983	-0.36	147.0441、157.0099、98.9746	[M+Na] +
38	D- (-)-Quinic acid	$C_7H_{12}O_6$	191.0562	5.144	0.45	93.0350、81.0346、59.0139	[M-H]-
39	Caffeic acid	$C_9H_8O_4$	163.039	5.241	0.03	89.0388、63.0226、77.0384	[M+H-H_2O] +
40	Cryptochlorogenic acid	$C_{16}H_{18}O_9$	353.087	5.334	-2.27	173.0453、179.0344、191.0555、135.0457	[M-H]-
41	Daphnetin	$C_9H_6O_4$	179.0339	5.466	0.20	68.9955、95.0637、93.0397	[M+H] +
42	6-Methoxyluteolin	$C_{16}H_{12}O_6$	359.076	5.501	-1.36	242.0583、271.0971、109.0296	[M+CH_3COO]-
43	4,4‘-Dihydroxydiphenyl Ether	$C_{12}H_{10}O_3$	220.0969	5.536	0.26	91.0548、115.0535、218.0800	[M+NH_4] +
44	Bergaptol	$C_{11}H_6O_4$	247.0247	5.584	-0.37	145.0295、173.0237、117.0343	[M+HCOO]-
45	Formononetin	$C_{16}H_{12}O_4$	286.1074	5.605	0.02	162.0550、79.0547、	[M+NH_4] +

						116.0497	
46	Deacetyl asperulosidic acid	$C_{17}H_{24}O_{11}$	427.1203	5.674	-1.76	98.0611、268.1433、322.1432	[M+Na] +
47	4‘-Hydroxyacetophenone	$C_8H_8O_2$	135.0453	5.682	1.34	108.0217、89.0397	[M-H]-
48	Neoeriocitrin	$C_{27}H_{32}O_{15}$	597.1736	5.760	1.12	525.1381、561.1588、459.1341	[M+H] +
49	Naringenin-6,8-di-C-glucoside	$C_{27}H_{32}O_{15}$	595.1662	5.801	-1.08	355.0823、385.0929、415.1035	[M-H]-
50	Morroniside	$C_{17}H_{26}O_{11}$	429.1366	5.967	-0.39	351.1033、369.1154、255.0655	[M+Na] +
51	Daidzein	$C_{15}H_{10}O_4$	253.0514	6.043	3.16	133.0302、135.0466	[M-H]-
52	Kuwanon	$C_{25}H_{26}O_6$	421.1629	6.181	-6.65	229.0129、285.0394、301.0356	[M-H]-
53	Coclaurine	$C_{17}H_{19}NO_3$	286.144	6.261	0.95	107.0491、115.0540、108.0524	[M+H] +
54	Myricetin-3-O-xyloside	$C_{20}H_{18}O_{12}$	449.0728	6.492	0.45	259.0615、287.0560、151.0012	[M-H]-
55	Eupatilin	$C_{18}H_{16}O_7$	343.0823	6.596	-0.15	135.0458、119.0501、184.0526	[M-H]-
56	Vicenin II	$C_{27}H_{30}O_{15}$	595.1653	6.641	-0.68	322.0747、391.0907、481.1195	[M+H] +
57	Lucenin-3	$C_{26}H_{28}O_{15}$	581.1495	6.935	-2.93	545.1272、443.0977、425.0876	[M+H] +
58	4,5-Dicaffeoylquinic acid	$C_{25}H_{24}O_{12}$	515.1189	6.942	-1.17	191.0563、135.0448、179.0035	[M-H]-
59	Myricetin	$C_{15}H_{10}O_8$	319.0424	7.073	-3.45	153.0187、245.0520	[M+H] +
60	Demethylzeylasteral	$C_{29}H_{36}O_6$	481.2618	7.487	4.16	147.0436、119.0493	[M+H] +
61	Complanatoside B	$C_{33}H_{40}O_{20}$	779.1997	7.557	-1.04	477.1566、331.1024	[M+Na] +

62	Camelliaside A	$C_{33}H_{40}O_{20}$	757.2183	7.583	-0.33	303.0499、85.0283、71.0489	[M+H] +
63	Alcesefoliside	$C_{33}H_{40}O_{20}$	755.2032	7.583	-1.1	301.0309、271.0248、255.0299	[M-H]-
64	Vicenin I	$C_{26}H_{28}O_{14}$	565.1545	7.695	-1.24	307.0577、379.0803、391.0795	[M+H] +
65	N-trans-p-Coumaroyloctopamine	$C_{17}H_{17}NO_{4}$	300.1231	7.713	0.35	116.0496、176.0707、144.0449	[M+H] +
66	Myricetin-3-O-galactoside	$C_{21}H_{20}O_{13}$	479.0827	7.825	-0.92	316.0209、271.0255、317.0261、287.0186	[M-H]-
67	2,6-Dimethoxyphenol	$C_{8}H_{10}O_{3}$	155.0704	7.973	3.22	91.0531、99.0434	[M+H] +
68	Quercetin-3-O-D-glucosyl-(1-2)-L-rhamnoside	$C_{27}H_{30}O_{16}$	609.1453	8.187	-1.39	300.0276、301.0309、271.0307	[M-H]-
69	Grosvenorine	$C_{33}H_{40}O_{19}$	763.2052	8.196	-0.52	477.1575、331.0995	[M+Na] +
70	Robinin	$C_{33}H_{40}O_{19}$	739.2085	8.205	-0.09	284.0326、285.0360、255.0299	[M-H]-
71	Clitorin	$C_{33}H_{40}O_{19}$	741.2236	8.213	-0.11	287.0551、85.0282、288.0583	[M+H] +
72	Kaempferol 3-rutinoside	$C_{27}H_{30}O_{15}$	595.1655	8.317	-0.47	287.0547、85.0285、71.0489	[M+H] +
73	Kaempferol	$C_{15}H_{10}O_{6}$	287.0552	8.386	0.56	153.0187、167.1092	[M+H] +
74	6,8-di-C-beta-D-xylopyranoside	$C_{25}H_{26}O_{13}$	535.1445	8.438	-0.27	409.0913、379.0805、445.0897	[M+H] +
75	Kaempferol-3-O-sophoroside	$C_{27}H_{30}O_{16}$	633.1423	8.654	-0.53	331.1005、325.0309、324.0234	[M+Na] +
76	Rutin	$C_{27}H_{30}O_{16}$	611.1605	8.731	-0.23	167.0715、87.0437、304.0641	[M+H] +
77	Quercetin	$C_{15}H_{10}O_{7}$	301.0366	8.809	3.99	245.0087、	[M-H]-

						83.0132、121.0321	
78	Genistein	$C_{15}H_{10}O_5$	271.0609	8.856	2.95	107.0867、197.8706	[M+H] +
79	Glucosyl-vitexin	$C_{27}H_{30}O_{15}$	617.1476	8.862	-0.18	331.0999、309.0364、185.0424	[M+Na] +
80	Gallic acid	$C_7H_6O_5$	169.0155	8.890	4.24	125.0236、107.0087	[M-H]-
81	Picroside II	$C_{23}H_{28}O_{13}$	535.1445	8.922	4.22	348.0634、336.0607、320.0658	[M+Na] +
82	6"-O-Acetylglycitin	$C_{24}H_{24}O_{11}$	533.1298	8.948	0.57	383.0769、353.0669、297.0751	[M+HCOO]-
83	Kaempferol-3-O-neohesperidoside	$C_{27}H_{30}O_{15}$	595.1655	8.991	-0.37	205.0476、86.0312、323.0601	[M+H] +
84	Luteolin	$C_{15}H_{10}O_6$	287.0548	9.008	-0.70	153.0182、121.0284	[M+H] +
85	Isoquercetin	$C_{21}H_{20}O_{12}$	463.0878	9.069	-0.87	300.0279、271.0251、255.0296	[M-H]-
86	Isoquercitrin	$C_{21}H_{20}O_{12}$	487.0844	9.077	-0.52	324.0220、335.0914、73.0281	[M+Na] +
87	Massoniresinol	$C_{20}H_{24}O_8$	415.1363	9.164	-0.11	89.0608、85.0264、73.0287	[M+Na] +
88	Luteolin-3-D-glucuronide	$C_{21}H_{18}O_{12}$	463.0869	9.285	-0.34	287.0542、288.0606、135.0792	[M+H] +
89	Vitexin-4’-O-glucoside	$C_{27}H_{30}O_{15}$	617.1474	9.302	-0.47	331.1405、338.9240、276.9989	[M+Na] +
90	Apigenin 6-C-α-L-arabinopyranosyl-8-C-β-D-xylopyranoside	$C_{25}H_{26}O_{13}$	535.1448	9.406	0.30	391.0816、325.0721、361.0730	[M+H] +
91	Loganin	$C_{17}H_{26}O_{10}$	449.1657	9.431	-1.65	155.1080、75.0080、59.0137	[M+CH_3COO]-
92	Quercitrin	$C_{21}H_{20}O_{11}$	471.0897	9.683	-0.26	308.0694、96.9952、328.0374	[M+Na] +

93	Kaempferol-3-glucorhamnoside	$C_{27}H_{30}O_{15}$	595.1652	9.769	-0.88	244.1437、97.0273、399.1418	[M+H] +
94	Salicylic acid	$C_7H_6O_3$	137.0245	10.053	0.64	93.0314、65.0407、75.0227	[M-H]-
95	Homoorientin	$C_{21}H_{20}O_{11}$	471.0895	10.099	-0.58	308.0296、309.0323、185.0414	[M+Na] +
96	Fisetin	$C_{15}H_{10}O_6$	287.055	10.116	0.14	153.0182、68.9971	[M+H] +
97	Astragalin	$C_{21}H_{20}O_{11}$	449.1076	10.185	-0.52	287.0544、303.0499、288.0599	[M+H] +
98	10-gingerol	$C_{21}H_{34}O_4$	351.2509	10.605	-2.36	137.0235	[M+H] +
99	Epicatechin	$C_{15}H_{14}O_6$	291.0845	10.861	-5.15	111.0481	[M+H] +
100	3,5-Dicaffeoylquinic acid	$C_{25}H_{24}O_{12}$	515.1189	10.882	-1.17	173.0448、191.0560、135.0445	[M-H]-
101	Rosmarinic acid	$C_{18}H_{16}O_8$	359.0765	10.934	-1.96	161.0239、72.9931、135.0455、133.0296	[M-H]-
102	L-Menthol	$C_{10}H_{20}O$	174.1853	11.224	0.23	55.0542、57.0692、93.0690	[M+NH_4] +
103	Madecassoside	$C_{48}H_{78}O_{20}$	1019.505	12.524	-1.63	469.1563、503.3381、161.0449	[M+HCOO]-
104	Glutamic acid	$C_5H_9NO_4$	148.0608	12.796	2.70	130.0649、56.5406	[M+H] +
105	Carveol	$C_{10}H_{16}O$	135.1164	13.523	-2.22	91.0541、107.0491	[M+H-H_2O] +
106	Ferulic acid	$C_10H_{10}O_4$	217.0468	13.626	-1.67	128.0632、177.1049	[M+Na] +
107	Umbelliferone	$C_9H_6O_3$	163.0392	13.740	1.23	77.0386、92.0257、51.0229	[M+H] +
108	Protocatechuic acid	$C_7H_6O_4$	153.0206	14.995	4.60	108.0217、65.0033、109.0295	[M-H]-
109	Pedunculoside	$C_{36}H_{58}O_{10}$	673.3922	15.215	-0.83	185.0444、511.3418	[M+Na] +

110	Nardosinone	$C_{15}H_{22}O_3$	251.1639	15.852	-0.98	67.0542、79.0542、81.0699	[M+H] +
111	8-Gingerol	$C_{19}H_{30}O_4$	323.2214	16.217	-0.96	277.2162、205.1223、305.2111	[M+H] +
112	Dihydroartemisinin	$C_{15}H_{24}O_5$	343.1759	16.655	-0.94	72.9930、207.1754、110.0364	[M+CH$_3$COO]-
113	Syringic acid	$C_9H_{10}O_5$	197.0459	17.175	1.78	153.0296、182.0018	[M-H]-
114	Dehydroeffusol	$C_{17}H_{14}O_2$	268.1342	17.444	3.73	55.9342、160.9167、72.9288	[M+NH$_4$] +
115	Tricin	$C_{17}H_{14}O_7$	331.2976	17.720	-0.96	333.1488、332.1922	[M+H] +
116	Undulatoside A	$C_{16}H_{18}O_9$	355.1033	18.065	2.25	175.1412、193.1232	[M+H] +
117	6-Shogaol	$C_{17}H_{24}O_3$	277.1798	18.273	-0.15	137.0597、55.0178、109.0648	[M+H] +
118	Sclareol Glycol	$C_{16}H_{30}O_2$	272.2587	19.086	0.96	57.0693、91.0543、104.0704	[M+NH$_4$] +
119	Dihydroxy-methoxy-methyl homoisoflavanone	$C_{18}H_{18}O_5$	337.1047	20.550	-1.00	209.1649、69.0690、70.0735	[M+Na] +
120	Myricitrin	$C_{21}H_{20}O_{12}$	465.1028	22.211	0.86	319.9724	[M+H] +
121	Dimethylophiopogonanone	$C_{20}H_{22}O_5$	365.1356	22.628	-0.72	91.0531、119.0855	[M+Na] +
122	2-Methoxycinnamaldehyde	$C_{10}H_{10}O_2$	163.0753	22.733	-0.61	77.0386、51.0229	[M+H] +
123	Triptophenol	$C_{20}H_{24}O_3$	311.1683	23.152	6.43	183.0088、119.0502	[M-H]-
124	Kaempferide	$C_{16}H_{12}O_6$	301.0707	23.300	1.99	161.9449、229.9486	[M+H] +
125	Dibutyl phthalate	$C_{16}H_{22}O_4$	301.1411	23.352	0.20	102.0322、55.0540	[M+Na] +
126	Tamarixetin	$C_{16}H_{12}O_7$	317.0650	27.470	-3.47	261.1704	[M+H] +
127	Isoschaftoside	$C_{26}H_{28}O_{14}$	565.1571	29.733	3.36	389.0598、353.1024、273.0406	[M+H] +
128	Guanosine	$C_{10}H_{13}N_5O_5$	284.0983	30.299	-2.11	110.0932	[M+H] +
129	Ethyl-p-methoxycinnamate	$C_{12}H_{14}O_3$	224.1280	31.920	-0.51	77.0383、	[M+NH$_4$] +

		79.0541、<br>91.0546	

Note: The compounds were putatively identified based on accurate mass, MS/MS fragmentation patterns, database matching, and literature reports. For compounds without confirmation by authentic standards or NMR data, exact linkage or substitution positions should be interpreted with caution.
